# Multi-System Inflammatory Syndrome in Children (MIS-C) Following SARS-CoV-2 Infection: Review of Clinical Presentation, Hypothetical Pathogenesis, and Proposed Management

**DOI:** 10.3390/children7070069

**Published:** 2020-07-01

**Authors:** Natasha A. Nakra, Dean A. Blumberg, Angel Herrera-Guerra, Satyan Lakshminrusimha

**Affiliations:** 1 Department of Pediatrics, Division of Infectious Diseases, University of California Davis School of Medicine, Sacramento, CA 95817, USA; dablumberg@ucdavis.edu; 2 Department of Pediatrics, Division of Allergy, Immunology, and Rheumatology, University of California Davis School of Medicine, Sacramento, CA 95817, USA; angelherrera@ucdavis.edu; 3 Department of Pediatrics, Division of Neonatology, University of California Davis School of Medicine, Sacramento, CA 95817, USA; slakshmi@ucdavis.edu

**Keywords:** SARS-CoV-2, COVID-19, multisystem inflammatory syndrome in children (MIS-C), Kawasaki Disease, toxic shock syndrome, hemophagocytic lymphohistiocytosis, macrophage activation syndrome

## Abstract

Severe acute respiratory syndrome coronavirus 2 (SARS-CoV-2) infection may result in the multisystem inflammatory syndrome in children (MIS-C). The clinical presentation of MIS-C includes fever, severe illness, and the involvement of two or more organ systems, in combination with laboratory evidence of inflammation and laboratory or epidemiologic evidence of SARS-CoV-2 infection. Some features of MIS-C resemble Kawasaki Disease, toxic shock syndrome, and secondary hemophagocytic lymphohistiocytosis/macrophage activation syndrome. The relationship of MIS-C to SARS-CoV-2 infection suggests that the pathogenesis involves post-infectious immune dysregulation. Patients with MIS-C should ideally be managed in a pediatric intensive care environment since rapid clinical deterioration may occur. Specific immunomodulatory therapy depends on the clinical presentation. The relationship between the immune response to SARS-CoV-2 vaccines in development and MIS-C requires further study.

## 1. Introduction

Severe acute respiratory syndrome coronavirus 2 (SARS-CoV-2) infection has rapidly spread worldwide since it was first identified in China in late 2019, with subsequent epicenters being recognized in Europe and the U.S. Previous reports of SARS-CoV-2 infection indicated that young children were disproportionately spared from infection [1,2], although it remains unclear if this is due to a lack of detection because of predominantly asymptomatic or mild disease in this age group. In the latter half of April 2020, a novel syndrome in children and adolescents termed “multisystem inflammatory syndrome in children” (MIS-C) with likely relation to SARS-CoV-2 infection was first described. Initial reports surfaced in the UK [3] and Italy [4], followed by New York and other parts of the U.S. Preliminary accounts of the features of this syndrome resemble those of known entities such as Kawasaki Disease (KD), toxic shock syndrome (TSS), and secondary hemophagocytic lymphohistiocytosis (SHLH)/macrophage activation syndrome (MAS). Here we review the preliminary data regarding the clinical presentation and complications of MIS-C, compare MIS-C to KD, TSS, and SHLH, and propose a plan for the evaluation and management of these patients.

## 2. Definition of MIS-C

**Figure 1:**
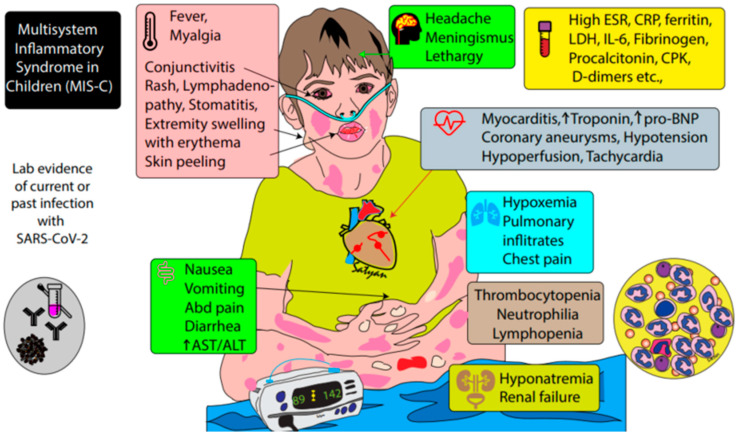
Infographic showing CDC criteria for the diagnosis of MIS-C. A combination of fever, evidence of inflammation, involvement of at least two organ systems, and prior evidence of SARS-CoV-2 infection are required to establish the diagnosis.

On 13 May, the Centers for Disease Control (CDC) issued a health advisory establishing the following definition for a reportable case of MIS-C (see Box 1 and Figure 1 for details) [5]:

Box 1 **CDC case definition for multisystem inflammatory syndrome in children (MIS-C).**

(1) An individual aged < 21 years with:
(2) Clinical criteria:
  - A minimum 24-h history of subjective or objective fever ≥ 38.0 °C AND
  - Severe illness necessitating hospitalization AND
  - Two or more organ systems affected (i.e., cardiac, renal, respiratory, hematologic, gastrointestinal, dermatologic, neurological)
(3) Laboratory evidence of inflammation
  - One or more of the following: an elevated CRP, ESR, fibrinogen, procalcitonin, D-dimer, ferritin, LDH, or IL-6; elevated neutrophils or reduced lymphocytes; low albumin
(4) Laboratory or epidemiologic evidence of SARS-CoV-2 infection
  - Positive SARS-CoV-2 testing by RT-PCR, serology, or antigen OR
  - COVID-19 exposure within 4 weeks prior to onset of symptoms
(5) No alternative diagnosis

Abbreviations: CDC, Centers for Disease Control; CRP, C-reactive protein; ESR, erythrocyte sedimentation rate; LDH, lactate dehydrogenase; RT-PCR, reverse transcriptase polymerase chain reaction; SARS-CoV-2, severe acute respiratory syndrome coronavirus-2.

## 3. Clinical Presentation of MIS-C and Patient Outcomes

Data regarding the clinical presentation and epidemiologic characteristics of children with MIS-C are still limited and evolving daily. In a case series from the UK [3], Italy [4], France, and Switzerland [6,7], the ages of affected children (*n* = 70) ranged from 2–16 years, and the majority did not have underlying comorbidities. Most had fever present for ≥4 days, and common presenting symptoms included gastrointestinal symptoms (59/70 = 84%), including vomiting, abdominal pain, and/or diarrhea; mucocutaneous symptoms reminiscent of KD, including conjunctivitis and rash; and neurologic findings including headache, irritability, and encephalopathy. A few children presented with an acute surgical abdomen and underwent exploratory laparotomy, with intra-operative findings of mesenteric lymphadenitis and peritonitis. Several children developed hypotension (52/70 = 74%) requiring admission to pediatric intensive care unit (PICU) and inotropic support, and some required non-invasive or invasive mechanical ventilation due to respiratory distress from cardiac dysfunction. A minority of children (11/70 = 16%) were placed on extra-corporeal membrane oxygen (ECMO) support. Echocardiography demonstrated depressed cardiac ventricular function in the majority of patients, but they were less commonly reported to have valvular regurgitation, dilated coronary arteries (11/70 = 16%). or frank coronary artery aneurysms (CAAs) (3/70 = 4%). Universally, laboratory testing revealed the significant elevation of inflammatory markers, such as C-reactive protein (CRP), erythrocyte sedimentation rate (ESR), procalcitonin, and/or ferritin. Other common findings included hyponatremia, acute kidney injury, and hypoalbuminemia, and several patients had serous effusions (pleural, pericardial, and peritoneal), suggestive of generalized inflammation. Troponin levels were elevated in many patients (57/70, 81%), and pro-B-type natriuretic peptide (proBNP) levels were markedly elevated in most (59/65 = 70%), suggesting myocardial damage and heart failure, respectively. Hematologic abnormalities reported included neutrophilia, lymphopenia, low to normal platelet levels, elevated D-dimer, and low fibrinogen. Thrombotic events were not reported.

The majority of affected children were treated with intravenous immune globulin (IVIG), and several also received adjunctive high-dose steroids. Most responded favorably to therapy with an improvement in vital signs and cardiac dysfunction, and only a few children required additional therapies, such as anakinra (recombinant IL-1β antagonist) or a second dose of IVIG. One child in the UK cohort was reported to develop a giant CAA after discharge from the initial hospitalization. Overall mortality has been low, with a single death in the UK cohort (due to a cerebrovascular accident while on ECMO), and three reported deaths in New York City (NY Times) [8].

Of note, a case report from the U.S. described a six-month infant with positive SARS-CoV-2 reverse transcriptase polymerase chain reaction (RT-PCR) testing from a nasopharyngeal swab who met the classical criteria for KD without evidence of multisystem involvement [9]. She was treated with IVIG and high dose aspirin, as per KD guidelines, and quickly defervesced with the resolution of stigmata of KD. At this time, it is unclear if MIS-C with KD features is different from KD. A positive SARS-CoV-2 test does not necessarily indicate causality and could represent coincident infection [10].

## 4. Relationship of MIS-C to COVID-19 and Pathogenesis

**Figure 2:**
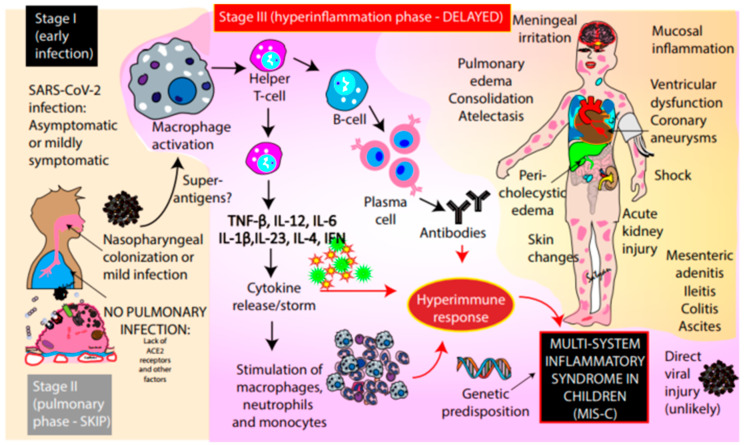
Pathogenesis of MIS-C. Early infection (phase I) with SARS-CoV-2 is likely to be asymptomatic or mildly symptomatic in children. The pulmonary phase (phase II) is severe in adults but is mild or absent in many children. The early infection appears to trigger macrophage activation followed by the stimulation of T-helper cells. This in turn leads to cytokine release, the stimulation of macrophages, neutrophils, and monocytes, along with B-cell and plasma cell activation with the production of antibodies leading to a hyperimmune response (stage III). This immune dysregulation is associated with the inflammatory syndrome in affected children. Direct infection with SARS-CoV-2 is less likely to play a role in MIS-C. ACE2—angiotensin converting enzyme 2 receptors; TNF-β—tumor necrosis factor β; IL—interleukins.

Epidemiologic evidence implicates SARS-CoV-2 as the likely cause of the newly recognized MIS-C, although causality has not yet been established (Figure 2). The emergence of clusters of cases in locations that have been heavily impacted by COVID-19, such as Italy, the UK, and New York City, is highly suggestive of a link to infection with SARS-CoV-2. The case series from Bergamo, Italy, a region with a high incidence of COVID-19 disease, described a 30-fold increase in the monthly incidence of KD cases between 18 February 2020 and 20 April 2020 in comparison to the previous 5 years [4]. On 13 May 2020, the New York State Department of Health (NYSDOH) reported 102 probable cases of MIS-C in New York hospitals, following the peak of COVID-19 infection in early April [11]. Interestingly, the cluster of MIS-C cases in these communities lags behind the peak COVID-19 incidence among adults by approximately one month. The fact that MIS-C was not identified in China and other Asian countries affected by COVID-19 has led to speculation regarding variations in the virus affecting areas with MIS-C cases or an increased susceptibility or genomic variation of these populations, although this is currently conjectural.

The majority of published cases have had positive serologic testing for SARS-CoV-2 (60/69, 87%) and less commonly positive RT-PCR testing from nasopharyngeal testing (23/70, 32%), suggesting that this syndrome may be post-infectious rather than related to acute early infection (Stage I—Figure 2). It is uncertain if broncho-alveolar lavage (BAL) sampling would increase the yield of detection in MIS-C. In adults with severe respiratory failure from SARS-CoV-2 infection, who typically experience clinical deterioration about 1 week following illness onset, a dysregulated immune system is thought to drive disease manifestations, as opposed to direct cellular injury from viral infection (Stage II—pulmonary phase). Children appear to have less severe pulmonary manifestations compared to adults, possibly due to lower gene expression of the angiotensin converting enzyme (ACE)-2 receptor (the target of SARS-CoV-2) [12,13]. Immune dysregulation in adults with respiratory disease is characterized by lymphopenia (specifically NK cells, CD4 T lymphocytes and B lymphocytes) and sustained production of pro-inflammatory cytokines, such as tumor necrosis factor (TNF)-α and interleukin (IL)-6 [14]. This immune dysregulation has been the basis of immunomodulatory therapies for adults with severe SARS-CoV-2 infection, such as tocilizumab, a humanized monoclonal antibody against the IL-6 receptor. In KD, a systemic hyper-inflammatory state is characterized by elevated levels of TNF, IL-6, IL-1β, IL-17, and granulocyte colony stimulating factor (G-CSF) [15]. We speculate that MIS-C is a delayed immunological phenomenon associated with inflammation (Stage III—hyperinflammation phase) following either symptomatic or asymptomatic COVID-19 infection.

## 5. Comparison of MIS-C with Known Syndromes

Similarities between patients with MIS-C and other well-defined syndromes, including KD, TSS, and SHLH/MAS, allow for hypotheses to be made regarding pathogenesis and may help guide treatment. Table 1 compares common clinical and laboratory findings between MIS-C, KD, and TSS.

### 5.1. Kawasaki Disease (KD)

KD is an acute medium-vessel vasculitis with a predilection for coronary arteries that occurs more commonly in young children; it is the most common cause of acquired heart disease in childhood in developed countries [16]. The KD defining features include rash, cervical lymph node enlargement, and ocular and oral mucosal changes, although the involvement of other organs, such as the liver, lungs, gastrointestinal tract, the central nervous system, and joints is widely recognized. Similar to MIS-C, laboratory markers of inflammation, such as CRP, are increased. Hematologic abnormalities are slightly different, as patients with KD tend to have leukocytosis with neutrophil predominance and thrombocytosis. Thrombocytopenia has been described in KD but is rare. The cardiac findings in MIS-C also are divergent from KD, as MIS-C patients are much more likely to exhibit cardiac dysfunction and hypotension, as opposed to coronary artery abnormalities [17].

Despite extensive efforts to identify the triggers for the inflammatory cascade in KD, the etiologic agent remains elusive. However, there is evidence that viral infections may elicit an inflammatory response in genetically predisposed children [18]. A viral trigger seems likely based on the typical occurrence of cases during winter and spring when respiratory viruses are circulating, as well as the young age of affected children, who are susceptible due to a lack of pre-existing immunity. Previous studies have attempted to implicate other human coronaviruses as the etiology of KD [19], but further studies did not confirm this association. The management of children with KD includes the administration of intravenous immune globulin (IVIG), treatment with high-dose aspirin, and occasionally the use of other immune-modulating drugs. The mechanism of IVIG is unclear, and may include immunomodulatory effects on T regulatory cells [20]. The treatment of children with KD has decreased the risk of CAAs from 25% to 4% [16].

A subset of children (<5%) with KD will present with shock/hypotension resembling bacterial sepsis. As compared to other children with KD, patients with “Kawasaki disease shock syndrome” (KDSS) have higher band counts, lower platelet counts, lower hemoglobin levels, and higher C-reactive protein levels [21]. They are also more likely to have coronary artery dilation and abnormalities of cardiac ventricular function [22]. The treatment approach to patients with KD and KDSS is similar, although patients with KDSS have higher rates of treatment failure with first-line therapies [23]. This subset of KD patients has several similarities to the newly described MIS-C.

### 5.2. Toxic Shock Syndrome (TSS)

TSS is a unique syndrome secondary to the uncontrolled activation of the immune system by “superantigens”, proteins that non-selectively stimulate T cells, resulting in massive cytokine release. Bacterial species, such as *Staphylococcus aureus* and *Streptococcus pyogenes*, are known to produce exotoxins that can function as superantigens, although viruses can also act as superantigens. Interestingly, prior research done on SARS-CoV-1 indicated that the viral structure included motifs consistent with superantigens [24]. The clinical presentation of toxic shock syndrome includes hypotension, diffuse erythrodermic rash, mucous membrane involvement, and multisystem organ dysfunction (renal, hepatic, hematologic, respiratory, muscular, and neurologic) [25]. Typical treatment for TSS includes volume resuscitation, treatment with anti-microbial agents directed against the inciting infection, and occasionally the use of IVIG for patients with refractory hypotension [26]. The proposed mechanisms of IVIG in patients with TSS include the neutralization of bacterial superantigens and the downregulation of the overactive immune response.

TSS can resemble KDSS, but patients with TSS tend to be older than those with KDSS (9 ± 4.6 years vs. 3 ± 3.4 years, respectively) [27] and are more likely to have normal hemoglobin, a lower platelet count, and elevated creatinine compared with KDSS patients. Patients with KDSS are more likely to exhibit coronary artery changes, valvulitis, and impaired cardiac ventricular function on echocardiogram as compared with TSS [27].

### 5.3. Secondary Hemophagocytic Lymphohistiocytosis/Macrophage Activation Syndrome (SHLH/MAS)

Hemophagocytic lymphohistiocytosis (HLH) is characterized by a robust immune response that is unabated and self-perpetuated. Primary HLH is secondary to anomalies in genes that regulate the degranulation of natural killer cells and cytotoxic CD8+ lymphocytes. This results in the inability to eliminate the antigenic stimuli that led to cellular activation, leading to a “cytokine storm” [28,29]. Elevated levels of pro-inflammatory cytokines, such as interferon (IFN)-gamma, IL-18, and IL-1, subsequently activate other cells of the immune system (i.e., macrophages) leading to organ damage and the characteristic hemophagocytosis of affected organs [29]. HLH is considered secondary when it is triggered by an autoimmune or autoinflammatory condition (referred to as MAS in this context), medications, malignancy, or infections. Regarding the latter, viral infections are well-known triggers of SHLH [30,31]. KD has also been associated with the development of MAS [32]. Notably, some patients that are thought to have SHLH are found to have mutations seen in primary HLH, so the distinction between primary and secondary HLH may be blurred [33].

Although not pathognomonic, the presence of hyperferritinemia (>500 ng/mL) should alert a physician to the possible presence of SHLH/MAS, especially in the presence of fever [34,35]. Patients with SHLH typically have evidence of systemic inflammation with elevated levels of CRP, triglycerides, and D-dimer, as well as organ dysfunction, such as coagulopathy, liver failure, CNS dysfunction, and cardiac dysfunction. Notably, the peripheral white blood cell count, platelet count, and ESR tend to be depressed in SHLH [29,34]. Mortality is high in untreated SHLH. Although not designed for infection-related SHLH, the criteria for primary HLH (Table 2) [36] or MAS in systemic juvenile idiopathic arthritis [37] may aid in the diagnosis of SHLH. It is important to note that the primary HLH criteria have low sensitivity for MAS [38] and this may be true for infection-associated SHLH as well. Additionally, the absence of hemophagocytosis in a bone marrow aspirate does not rule out the diagnosis.

## 6. Proposed Clinical Evaluation of Suspected MIS-C

Patients with MIS-C may quickly progress to critical illness and hypotension. Therefore, they should be managed in a center with pediatric intensive care capabilities. Laboratory evaluation for generalized inflammation, multisystem involvement, and possible infection is appropriate (Box 2). Particular attention should be paid to the monitoring of cardiac function, respiratory status, neurologic status, and renal function. Depending on organ system involvement, the early consultation of specialists from pediatric intensive care, cardiology, rheumatology, infectious disease, immunology, and neurology should be considered.

Box 2 **Proposed approach to children presenting with signs concerning for MIS-C.**

1. Consider observation in unit with cardio-respiratory monitoring capabilities
2. Laboratory evaluation
  - Complete blood count with differential
  - Blood chemistry, including BUN and creatinine
  - Liver function tests (ALT, AST, albumin, bilirubin)
  - Cardiac markers: troponin and pro-BNP
  - Urinalysis with culture if indicated
  - Blood gas with lactate
  - Markers of inflammation: ESR, CRP, procalcitonin, ferritin, triglycerides, IL-6 if available
  - Coagulation panel: PT, PTT, fibrinogen, D-dimer
  - Creatinine kinase, lactate dehydrogenase
  - Blood culture
  - Serology for SARS-CoV-2
  - NP swab or lower respiratory tract sample for SARS-CoV-2 by RT-PCR; consider sending from stool if presenting with GI symptoms
  - Additional studies as indicated: respiratory pathogen panel from NP swab or lower respiratory tract, stool studies/cultures, viral blood PCRs or serologies to rule out other causes of myocarditis, genetic testing for HLH, soluble IL-2 receptor, NK cell function
3. Imaging:
  - Chest X-ray
  - Abdominal ultrasound or CT scan if concerning symptoms/physical findings
4. Twelve-lead electrocardiogram (EKG)
5. Echocardiogram (transthoracic)
6. Early consultation of specialists to assist in management, such as intensive care, cardiology, rheumatology, infectious diseases, allergy/immunology, neurology

Abbreviations: ALT, alanine transaminase; AST, aspartate transaminase; pro-BNP, pro-B-type natriuretic peptide; BUN, blood urea nitrogen; CRP, C-reactive protein; CT, computed tomography; ESR, erythrocyte sedimentation rate; GI, gastrointestinal; HLH, hemophagocytic lymphohistiocytosis; IL, interleukin; MIS-C, multisystem inflammatory syndrome in children; NK, natural killer; NP, nasopharyngeal; PT, prothrombin time; PTT, partial thromboplastin time; RT-PCR, reverse transcriptase polymerase chain reaction; SARS-CoV-2, severe acute respiratory syndrome coronavirus 2.

Of note, in the absence of a positive test for SARS-CoV-2 or a positive epidemiological exposure, we suggest the consideration of alternative diagnoses. Serology can be repeated 2–4 weeks later, and if results are negative but suspicion remains high, serology can be repeated using a different assay. The testing of household contacts can also reveal evidence of exposure.

## 7. Suggested Management of Patients with MIS-C

The goals of treatment for MIS-C are to decrease systemic inflammation and restore organ function, in order to decrease mortality and reduce the risk of long-term sequelae, such as the development of CAAs or persistent cardiac dysfunction. Treatment should be dictated by phenotype until new research provides clear guidelines. Given the novelty of this syndrome, the following recommendations are based on extrapolation from other syndromes and constitute the opinion of the authors (See Table 3 for detailed dosing of medications).

### 7.1. Clinical Features Consistent with KD

All patients meeting the criteria for KD should be treated as per published guidelines. First-line therapy for KD includes treatment with high-dose IVIG and aspirin. Given the highly favorable results in early case series of children with MIS-C who received IVIG, we would also recommend the consideration of IVIG for patients with MIS-C who do not meet KD criteria for possible beneficial immunomodulatory effects, analogous to its use in TSS.

Corticosteroid treatment is a commonly used adjunctive therapy to IVIG for treatment of KD. In Japan, scoring systems such as the Kobayashi score predict a high risk of IVIG resistance and have been used to inform decisions about who should receive steroids concomitantly with IVIG [41]. This approach has been shown to decrease the risk of coronary abnormalities in high-risk children, and could be helpful in to inform treatment decisions in MIS-C [42,43]. Per KD guidelines from the European initiative Single Hub and Access point for paediatric Rheumatology in Europe (SHARE) [39], corticosteroids should be considered for children with features of severe KD, defined by fever or persistent inflammation ≥ 48 h after IVIG, Kobayashi score ≥ 5, features of SHLH (i.e., ferritin > 500 ng/mL), shock, age < 1 year, or coronary or peripheral aneurysms at the time of diagnosis.

Therapy with anakinra may be considered for patients with KD-like disease who are refractory to first-line therapy with IVIG (with or without the addition of corticosteroids) [44]. Tocilizumab is an IL-6 inhibitor that has been used in the setting of refractory KD, although one report demonstrated the rapid development of CAAs in two out of four patients following therapy, suggesting caution be used with this agent in patients with KD [45]. It is important to note that the evidence for this association is weak and contradicts the favorable results obtained with tocilizumab in the treatment of large cell vasculitis in adults [46].

### 7.2. Clinical Features Consistent with SHLH

For the management of MIS-C with features of SHLH involvement, the consultation of pediatric rheumatology, immunology, or hematology/oncology is highly recommended. For initial treatment, we suggest following the cytokine storm treatment pathway described by Halyabar et al. [28,34] with anakinra and IVIG.

Tocilizumab (IL-6 inhibitor) has been studied for the treatment of COVID-19 infection in adults in an uncontrolled study [47]. Although no conclusions can be made from this study regarding the efficacy of tocilizumab in COVID-19 pneumonia, the survival rates reported are encouraging and suggest that tocilizumab is at least safe in severe COVID-19 infection. We consider that tocilizumab may also be substituted for anakinra in MIS-C with SHLH features, especially in the setting of active SARS-CoV-2 infection. For severe disease, the addition of pulsed corticosteroids and other immunosuppressive agents, such as cyclosporine and tacrolimus, may be considered. If MIS-C is truly a post-infectious process, the immunosuppressive effects of therapy would not risk the flare of infection, as the infection is resolved. However, in the case of active SARS-CoV-2 infection, there is the theoretical possibility that immunomodulatory treatment could worsen the infection. Anakinra has been proven to be effective and safe for the treatment of sepsis with features of SHLH [48]. Regarding the safety of steroids in the setting of active COVID-19 infection (SARS-CoV-2 RT-PCR positive), no conclusions can be made with the currently available evidence; steroids or other immunosuppressive agents may be warranted for particular cases. Pulsing with a lower dose of methylprednisolone (10 mg/kg/dose) may be an alternative for severe SHLH.

### 7.3. Outpatient Management of Children under Investigation for MIS-C

For some children with short duration of fever (<4 days) who are not critically ill and do not exhibit multi-system involvement, initial evaluation in the ambulatory setting may be considered. A preliminary laboratory workup, including complete blood count with differential, liver function tests, electrolytes, CRP, ESR, urinalysis, and SARS-CoV-2 nasopharyngeal PCR and serology can be helpful to decide whether further evaluation or hospitalization is necessary. Evaluation for other causes of fever as appropriate based on symptomatology (i.e., respiratory viruses, streptococcal pharyngitis) should be undertaken. For children with persistent fever or new symptoms, repeat clinical evaluation and laboratory testing should be performed in the ambulatory or emergency department (ED) setting every 24 h. Referral to a hospital providing specialty pediatric care should be strongly considered for abnormal laboratory values or changes in clinical status.

### 7.4. Other Considerations

Antiviral therapy with remdesivir, a nucleotide analogue with in vitro activity against SARS-CoV-2 [49], may be considered for SARS-CoV-2 RT-PCR positive patients. It is not likely to be of benefit for patients who are PCR negative, as studies have shown its benefit is greatest when administered early in disease [50]. Initial treatment with broad antimicrobials is appropriate, given that many of these patients present with clinical features and laboratory findings consistent with bacterial sepsis. However, we recommend that antimicrobial treatment be discontinued once a patient is recognized to have MIS-C and bacterial cultures are negative.

## 8. Future Directions

Elucidating the pathogenesis of MIS-C will be critical to inform rational management strategies and possible preemptive measures. More robust clinical data will be useful in determining risk factors for the development of MIS-C, as well as prognosis. The follow up of MIS-C patients is imperative for determining possible sequelae. Immunologic studies involving serial measurements of both cell-mediated and cytokine immune responses will provide insight into pathogenesis. The creation of a registry of MIS-C patients may be the most expedient manner to compile this information. Genetic studies will be vital to our understanding of why some children with SARS-CoV-2 infection ultimately develop MIS-C.

Of note, a widespread SARS-CoV-2 vaccine use could theoretically predispose to MIS-C, given that this is likely an immune-mediated phenomenon. The risk of MIS-C will depend on the qualities of the immune response achieved, for example, whether the Th1 or Th2 response is predominant [51]. As an example, initial attempts at a respiratory syncytial virus vaccine found that a formalin-inactivated vaccine candidate resulted in enhanced pulmonary disease after subsequent infection [52]. Some deaths were attributed to an intense Th2 vaccine response which resulted in profuse pulmonary inflammation and subsequent parenchymal tissue damage. This has implications for vaccine development, especially in the selection of adjuvants to generate the intended immune response [53]. An additional concern related to MIS-C and immunization is the effect of mutations in circulating SARS-CoV-2 strains [54] and whether heterotypic immunologic responses may result in severe immunologic manifestations after subsequent infection, similar to concerns regarding dengue vaccines [55]. The close monitoring of MIS-C incidence will be required, as immunization trials are carried out in children. The hope is that a fully effective vaccine will preempt MIS-C.

## 9. Conclusions

Based on initial reports from China, it was thought that children have a low incidence of symptomatic infection. However, the increased prevalence of MIS-C suggests a delayed hyperimmune response to SARS-CoV-2 infection. The exact incidence of MIS-C following an asymptomatic or mildly symptomatic infection with SARS-CoV-2 is not known. Further studies evaluating the predisposing factors and pathogenesis of MIS-C are warranted to appropriately prevent and optimally manage this condition.

## Figures and Tables

**Table 1:** Comparison of clinical and laboratory features of MIS-C with KD, KDSS, and TSS.

	Pediatric MIS-C	Kawasaki Disease (KD)	Kawasaki Disease Shock Syndrome (KDSS)	Toxic Shock Syndrome (TSS)
Age of affected children	Older (range 6 m–16 y)	Younger	Younger	Older
Hypotension	±	−	++	++
Mucous membrane involvement	±	+	+	±
Rash	+	+	+	Typically erythroderma
Desquamation	+	+	+	+
Altered mental status or encephalopathy	+	Rare	+	+
Vomiting, diarrhea, and/or abdominal pain	++	Rare	+	+
Respiratory distress	+	Rare	+	±
Myalgias	+	−	−	+
WBC differential	Neutrophilia, lymphopenia	Neutrophilia	Neutrophilia	Neutrophilia
Platelets	↓	↑	↓, normal, or ↑	↓
PT/PTT	↑	normal	normal or ↑	↑
Fibrinogen	↓, normal, or ↑	normal	normal, or ↑	↓
D-dimer	↑	normal	normal, or ↑	↑
ALT	normal, or ↑	normal, or ↑	normal, or ↑	normal, or ↑
Creatinine	↑	normal	↑	↑
Troponin	↑	normal, or ↑	↑	ID
Pro-BNP	⇈	normal, or ↑	↑	ID
Ferritin	↑	normal, or ↑	normal, or ↑	normal
CRP	⇈	↑	⇈	↑
Coronary artery dilation or aneurysms	+	+	++	−
Cardiac ventricular dysfunction	+	±	+	Rare
Valvular regurgitation	+	+	++	Rare

Abbreviations: +, generally present; ++, almost always present; −, generally absent; ±, may be present or absent; ↑ increased; ⇈, highly increased; ↓ decreased; ALT, alanine transaminase; pro-BNP, pro-B-type natriuretic peptide; CRP, C-reactive protein; ID, insufficient data; KD, Kawasaki Disease; KDSS, Kawasaki Disease shock syndrome; m, months; MIS-C, multisystem inflammatory syndrome in children; PT/PTT, prothrombin time and partial thromboplastin time; TSS, toxic shock syndrome; WBC, white blood cell count; y, years.

**Table 2:** 2004 diagnostic criteria for hemophagocytic lymphohistiocytosis (HLH) [36].

HLH Diagnosis Can Be Established with either 1 or 2.
Molecular diagnosis consistent with HLH Diagnostic criteria fulfilled by meeting five out of the eight criteria below Fever Splenomegaly Cytopenias affecting two out of three blood lineages in peripheral blood Hemoglobin < 90 g/L (in infants < 4 weeks: hemoglobin < 100 g/L) Platelets < 100 × 10^9^/L Neutrophils < 1.0 × 10^9^/L Hypertriglyceridemia and/or hypofibrinogenemia: Fasting triglycerides ≥ 3.0 mmol/L (i.e., ≥265 mg/dL) Fibrinogen ≤ 1.5 g/L Hemophagocytosis in bone marrow or spleen or lymph nodes and no malignancy Low or absent NK cell activity (according to local laboratory reference) Ferritin ≥ 500 mg/L Soluble CD25 (i.e., soluble IL-2 receptor) > 2400 U/mL

Abbreviations: dL, deciliter; g, grams; IL, interleukin; L, liter; mg, milligrams; mL, milliliter; mmol, millimoles; U, units.

**Table 3:** Possible doses for immunomodulatory agents in the treatment of MIS-C, depending on phenotypic characteristics.

Medication Class	Dose	Important Notes
IVIG [16,34]	If they meet KD criteria: 2 g/kg IV typically given in a single dose If they meet SHLH criteria: 1–2 g/kg IV	Use with caution if fluid overload, renal dysfunction. Consider alternate dosing strategy.
Aspirin	If they meet KD criteria: 30–50 mg/kg/d, decrease to 3–5 mg/kg/d once afebrile × 48 h	Precaution in severe thrombocytopenia
Corticosteroids [34,39]	For severe KD *: ○ Dosing strategy 1: Methylprednisone 0.8 mg/kg BID IV for 5–7 d or until CRP normalizes followed by PO prednisone/prednisolone 2 mg/kg/d with wean over 2–3 w ○ Dosing strategy 2: Methylprednisolone 10–30 mg/kg IV QD for 3 d followed by PO prednisone/prednisolone 2 mg/kg/d until d 7 or until CRP normalizes and then wean over 2–3 w For SHLH ** ○ Methylprednisone pulsed dosing of 30 mg/kg IV QD × 3 doses followed by 1 mg/kg IV q12 h, wean to be determined by peds rheumatology, immunology, or H/O	Precaution if positive RT-PCR for SARS-CoV-2, suggesting active infection
Anakinra [16,34]	2–6 mg/kg/day IV/SQ, length of therapy to be decided with input from pediatric rheumatology or immunology	
Tocilizumab	<30 Kg: 12 mg/kg IV >30 Kg: 8 mg/kg IV	Trials ongoing for safety and efficacy in the setting of active coronavirus infection [40]

Abbreviations: BID, twice daily; d, days; g, gram; h, hours; H/O, hematology–oncology; IV, intravenous; IG, immune globulin; KD, Kawasaki disease; kg, kilograms; mg, milligrams; PO, by mouth; q, every; QD, every day; RT-PCR, reverse transcriptase PCR; SHLH, secondary hemophagocytic lymphohistiocytosis; SQ, subcutaneous; w, weeks. *—see text for definition. **—per clinical discretion.
